# Global AIDS Reporting-2001 to 2015: Lessons for Monitoring the Sustainable Development Goals

**DOI:** 10.1007/s10461-016-1662-9

**Published:** 2017-01-25

**Authors:** T. Alfvén, T. Erkkola, PD Ghys, J. Padayachy, M. Warner-Smith, D. Rugg, P. de Lay

**Affiliations:** 1 Global Health - Health Systems and Policy, Department of Public Health Sciences, Karolinska Institutet, Solna, Sweden; 2 UNAIDS (The Joint United Nations Programme on HIV/AIDS), Geneva, Switzerland; 3 Cancer Institute, NSW Health, Sydney, NSW Australia; 4 Claremont Evaluation Center- New York, New York, USA; 5 Washington, DC, USA

**Keywords:** AIDS, GARPR, HIV, MDG, SDG, UNGASS, Monitoring, Evaluation

## Abstract

Since 2001 the UNAIDS Secretariat has retained the responsibility for monitoring progress towards global commitments on HIV/AIDS. Key critical characteristics of the reporting system were assessed for the reporting period from 2004 to 2014 and analyses were undertaken of response rates and core indicator performance. Country submission rates ranged from 102 (53%) Member States in 2004 to 186 (96%) in 2012. There was great variance in response rates for specific indicators, with the highest response rates for treatment-related indicators. The Global AIDS reporting system has improved substantially over time and has provided key trend data on responses to the HIV epidemic, serving as the global accountability mechanism and providing reference data on the global AIDS response. It will be critical that reporting systems continue to evolve to support the monitoring of the Sustainable Development Goals, in view of ending the AIDS epidemic as a public health threat by 2030.

**Electronic supplementary material:**

The online version of this article (doi:10.1007/s10461-016-1662-9) contains supplementary material, which is available to authorized users.

## Resumen

Desde el 2001, el Secretariado de ONUSIDA tiene la responsabilidad de monitorear los avances hacia los compromisos globales relacionados al VIH/SIDA. Características claves del sistema de reporte fueron examinadas para el periodo de reporte desde 2004 al 2014, y análisis fueron completados de las tasas de reporte y el rendimiento de cada indicador. La tasa de entrega de reportes de país varió desde 102 (53%) Estados Miembros en 2004 a 186 (96%) en 2012. Hubo gran variación en las tasas de respuesta de indicadores específicos, con los indicadores relacionados a tratamiento teniendo las tasas de reporte más altas. El sistema de reporte global sobre SIDA ha mejorado sustancialmente a través del tiempo y ha brindado datos claves de tendencias en la respuesta a la epidemia de VIH, sirviendo como mecanismo de rendición de cuentas y brindando datos de referencia sobre la respuesta global al SIDA. Será crítico que sistemas de reporte sigan evolucionando para apoyar el monitoreo de las Metas de Desarrollo Sostenible, con la visión de poner fin a la epidemia de SIDA para 2030.

## Introduction

Setting targets in international development became commonplace in the 1960s, the first *“UN Development Decade”*. Targets were set to address a range of development issues, ranging from education to food security and health. However, mechanisms for developing and implementing appropriate plans of action and for monitoring progress towards the targets were not established and results often fell far short of target levels. A significant change came with the UN World Summit for Children in 1990 when Jim Grant, then UNICEF (The United Nations Children’s Fund) Executive Director, along with colleagues and social activists set in motion the implementation and monitoring processes to instigate momentum behind the Summit’s Declaration: countries established national programs and conducted surveys using recognized, standardized indicators in an unprecedented way. The importance of ongoing follow-up and monitoring of UN commitments was shown to make critical differences in establishing national ownership, global financial support and overall accountability.

In 2000, 189 nations made a promise to free people from extreme poverty and to address a set of social and health concerns in the United Nations Millennium Declaration. This pledge evolved into the eight Millennium Development Goals (MDGs), of which MDG 6 is “Combat HIV/AIDS, malaria and other diseases” [1, 2].

In 2001, Member States of the United Nations along with civil society groups including people living with HIV, convened at a United Nations General Assembly Special Session (UNGASS) on HIV/AIDS. This led to 189 countries adopting a Declaration of Commitment (DoC) to intensify efforts to prevent HIV infection and to increase the coverage and quality of services for people living with and affected by HIV. In the DoC, time-bound and specific targets were set in selected priority areas with the intention of catalyzing intensified programming. The Declaration stipulated that progress should be reviewed every 2 years [3]. Following the adoption of the DoC, the UN Secretary General charged UNAIDS (The Joint United Nations Programme on HIV/AIDS) with the responsibility of monitoring its implementation in all countries in a biennial country reporting cycle.

Influenced by the availability of life-saving treatment for HIV and AIDS, in the 2006 Political Declaration on HIV/AIDS [4], UN Member States renewed their commitment to the Declaration of Commitment on HIV/AIDS issued in 2001 and agreed to work towards the broad goal of “universal access to comprehensive prevention programmes, treatment, care and support” by 2010.

The “2011 UN Political Declaration on HIV and AIDS: Intensifying our Efforts to Eliminate HIV and AIDS” (General Assembly resolution 65/277) was adopted at the United Nations General Assembly High Level Meeting on AIDS in June 2011, and mandated UNAIDS to support countries to report on the commitments of the declaration [5], which led to a change from a biennial to an annual country reporting cycle.

This paper identifies key characteristics that have contributed to the development of the Global AIDS Reporting System and describes progress in monitoring the AIDS epidemic and the response since the global commitments on AIDS in 2000 and 2001 and reviews national reporting rates and indicator performance.

## Methods

### Data Sources

Indicator definitions and guidance on the use of indicators were derived from UNGASS reporting guidelines in 2004, 2006, 2008 and 2010 and from Global AIDS Response Progress Reporting (GARPR) guidelines in 2012, 2013, 2014 and 2015 [6–13]

Reporting rates and core indicator performance were derived from country progress reports (all data submitted by the Member States, including both indicator data and country narrative reports) submitted to UNAIDS from 2004 to 2014.

In addition to the quantitative analyses, a qualitative review of the value, relevance, and accuracy of the indicators was performed through examination of the published literature on global AIDS reporting, in combination with results from evaluations on the reporting system, and through key informant interviews with UNAIDS staff at HQ, regional and country level.

### Evolution of Indicators Over Time

Short descriptions of all indicators used in global AIDS reporting since 2003 were extracted from UNGASS and GARPR guidelines [6–13] and an analysis undertaken of how and why the indicator set evolved over time.

### Reporting Rates

Reporting rates were calculated taking the number of Member States that submitted a Country Progress Report divided by the total number of Member States at the time of reporting.

### Indicator Performance Analysis

These analyses provide a summary of the performance of seven key indicators for Global AIDS reporting over time (2006 until 2015 reporting). The seven assessed indicators cut across the different key areas in the reporting system: *behavioural data*, sourced from population-based surveys [Knowledge about HIV prevention (GARPR indicator 1.1)], from key populations at higher risk of HIV, such as sex workers (condom use; GARPR indicator 1.8), men who have sex with men (MSM) (condom use; GARPR indicator 1.12), and people who inject drugs (prevention programmes; GARPR indicator 2.1); and indicators based on *programme data*, such as prevention of mother to child transmission (GARPR indicator 3.1) and HIV treatment—antiretroviral therapy (GARPR indicator 4.1); and lastly *policy related questions* from the National Commitments and Policy Instrument (NCPI). The indicator data were reported every second year until the 2011 reporting period, when the frequency was changed to annual progress reporting (except NCPI which continued to be reported biennially). In addition to their programmatic relevance, these seven indicators have stayed the most consistent over time (except for indicator 2.1), and were considered most representative of different epidemiological contexts.

## Results

Updated guidelines on the construction of the indicators have been made available in advance of each reporting round. These guidelines describe in detail the full indicator specifications and data collection methods to ensure consistent data across countries. They also provide guidance on the analysis of the indicator data for country use.

The indicator set has evolved over time (Online Appendix for a full table of all UNGASS and GARPR indicators over time). In 2002 a series of core indicators were developed and were grouped in four broad categories: (i) national commitment and action; (ii) national knowledge and behavior; (iii) national impact; and (iv) global commitment. This structure was kept until the 2012 reporting when they were restructured around ten targets and elimination commitments based on the 2011 UN Political Declaration on HIV and AIDS [5]. The 2015 indicator set includes 31 indicators, including national programmes (e.g. coverage of treatment and prevention services), HIV-related knowledge and behaviour, and the level of impact (e.g. HIV prevalence among young women). In addition to quantitative indicators, it also contains detailed data on domestic and international spending and the policy environment. Five of these indicators served to monitor the Millennium Development Goal (MDG) for AIDS, i.e. halting and reversing the AIDS epidemic by 2015.

The resulting strategic AIDS information system, referred to as the UNGASS Reporting System between 2003 and 2011 and the GARPR system since 2011, has been predicated on the submission of Country Progress Reports, including indicator data, biennially between 2004 and 2011 and annually since 2012.

## Key Characteristics of the Global AIDS Reporting System

Key characteristics of the Global AIDS reporting system over time are summarized below. The important issue of data sharing and transparency is summarized in text box no 1.

### Strong Technical Oversight and Harmonisation

In preparations for the first round of Global AIDS reporting in 2004, the UNAIDS Secretariat used the technical expertise of its Monitoring and Evaluation Reference Group (MERG), which included representatives from international agencies, national governments, civil society and academia, to select a concise set of existing indicators most relevant to the key components of a national HIV response and key epidemiological data. The MERG has continued to provide oversight over the system, leading reviews and proposing changes, e.g. when new programmes were introduced, such as interventions for the prevention of mother to child HIV transmission (PMTCT).

In order to reduce the reporting burden for countries, and facilitate global and regional work, the UNAIDS secretariat, through its global, regional and country offices, has worked closely with other reporting systems, such as the WHO health sector response reporting and the European Centre for Disease Prevention and Control (ECDC) Dublin Declaration reporting in Europe and Central Asia to streamline reporting processes through joint reporting and harmonization of indicators.

### Provision of In-Country Technical Support

In 2004, UNAIDS deployed technical field staff, who were mandated to support the development of systems for the production, analysis, interpretation and reporting of data on national HIV responses. They have been instrumental in assisting the country-based process and training country staff in the preparation and submission of Country Progress Reports. Initially called *Monitoring and Evaluation Adviser*, the staff function was expanded in 2011 to *Strategic information Adviser,* with greater focus on analysis and programmatic advice. In addition to this technical staff support, direct financial assistance was provided to “kick-start” the reporting process in low- and middle-income countries. Over time, this financial support was reduced as countries’ monitoring and reporting systems became increasingly self-sustaining. Furthermore each round has been supported by training workshops for international and national staff, initially as regional face-to-face workshops and then during the last four reporting rounds in most regions as web-based seminars.

### Clearly Documented Reporting Requirements

Guidelines on Global AIDS reporting have been regularly disseminated by UNAIDS prior to the country reporting deadlines. These guidelines recommend that each country conduct data needs assessments; develop data collation and reporting plans; establish data processing procedures (including cleaning, validation and data entry into a single database); conduct data vetting and data triangulation workshops to obtain consensus on the values to be reported. It was recommended that this process should involve a wide range of stakeholders including representatives from relevant government departments, civil society organizations and non-governmental organisations (NGOs), and international agencies (where applicable).

### A Comprehensive Consultation and Communications Strategy

UNAIDS communications and advocacy strategies at country level have proven to be important and have targeted three different audiences associated with reporting: political leaders, government and partner agency technical staff, and civil society. Each reporting round has started with a letter to Member States missions in Geneva and New York that explains the upcoming reporting, followed up with a more technical note to national monitoring and strategic information staff, usually based at Ministries of Health (MoH) or at National AIDS Councils/Commissions (NACs). Civil society was a driving force behind the 2001 DoC and has continued to be a very active and important partner at global, regional and national level [14].

### Sound Data Quality Assurance Mechanisms

UNAIDS Secretariat staff, together with colleagues from WHO and UNICEF and other international organisations, worked together with national staff to ensure the availability of high quality data. The Country Progress Reports received by the UNAIDS Secretariat are systematically checked for calculation errors, illogical values, and missing data fields. In many countries where UNAIDS staff are deployed this data quality assurance occurs first at country level, then moves up to regional level and finally at global level. When issues are detected, they are discussed and resolved with technical staff from the relevant country allowing continued country ownership of the data. Additional feedback is provided to countries on request to allow for further improvements in country M&E systems.

As part of data processing at the UNAIDS Secretariat level, key international agencies involved in global reporting on AIDS (such as UNAIDS, Global Fund, PEPFAR (President’s Emergency Plan for AIDS Relief), UNICEF, WHO) reconcile data in an effort to improve data accuracy and reduce discrepancies in reported figures at the global level.

### Regular Review of Indicators and Processes

The performance of the Global AIDS reporting indicators has been reviewed after each round of reporting and revised in response to technical issues, as well as new programmatic developments. As a result, reporting has continued to improve in both comprehensiveness and quality and, due to the active involvement of country representatives, has become more responsive to country needs [15]. In 2010, a major review of the reporting system and the indicators was executed by UNAIDS Secretariat and co-sponsors in tandem with civil society, using explicit guidelines for indicator quality enhancement [16] in order to update and refine the system when the UNGASS indicators became the Global AIDS Response Programme Reporting indicators (GARPR).

As per its mandate, UNAIDS supports annually the United Nations Secretary General in reporting to the General Assembly on progress towards the global targets. In addition, progress reporting has also been used to assess achievements against the MDG 6 target. Since 2003, to inform World AIDS Day (1st of December) activities or international AIDS conferences, UNAIDS has published reports in every reporting round. These reports serve as reference documents for many institutions, holding governments accountable for progress against global targets. Similarly, UNAIDS produces regional reports and analysis, such as through the AIDS data-hub in Asia Pacific [17], serving a similar purpose for regional organizations. All the country reports are published on UNAIDS web-site, and often also on national web-sites, increasing transparency and access to data. Since 2010, UNAIDS has published all the data on its data visualization tool, AIDSinfo, which receives over 35,000 individual visitors annually. For advanced analysis, the full data set is also made available in AIDSinfo online database. The tools enable public access to the global, regional and country data on HIV and AIDS. Other international development indicator datasets, such as those held by the UN statistics division, UNDP, WHO and the World Bank include indicator data from the UNAIDS dataset, allowing comparisons and cross analysis with other development issues and targets.

**Fig. 1:**
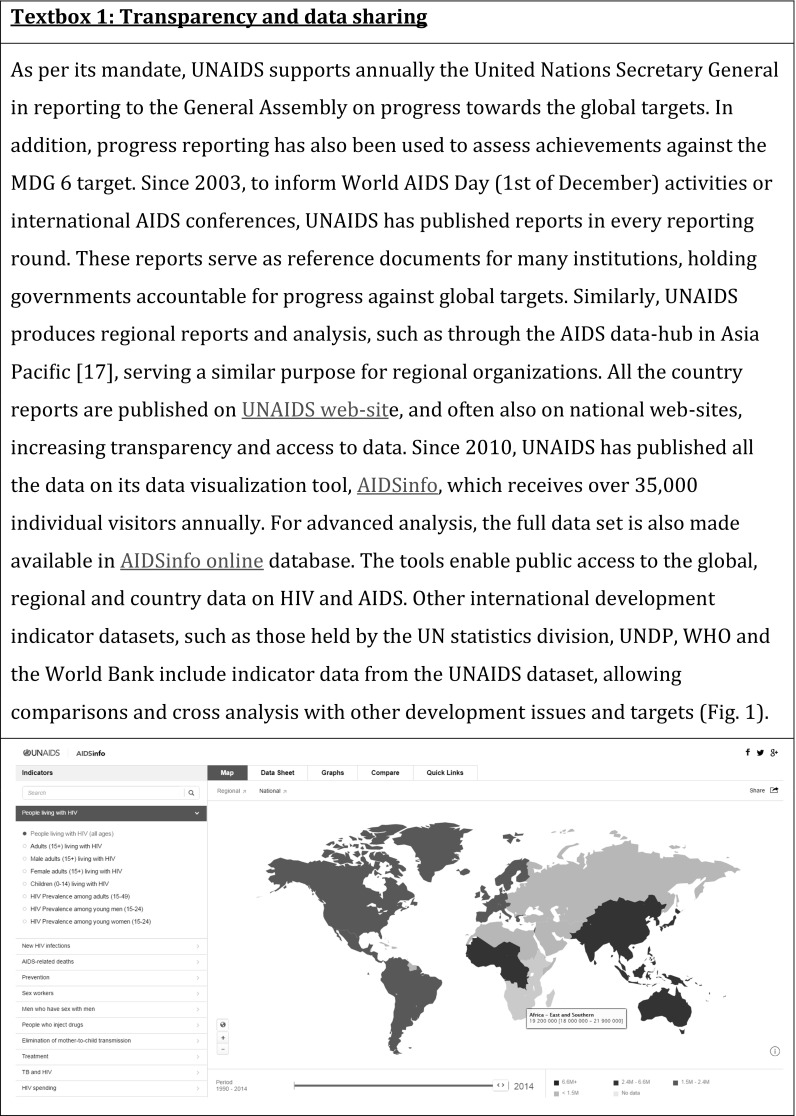
AIDSinfo database (Color figure online)

## Reporting Rates

**Fig. 2:**
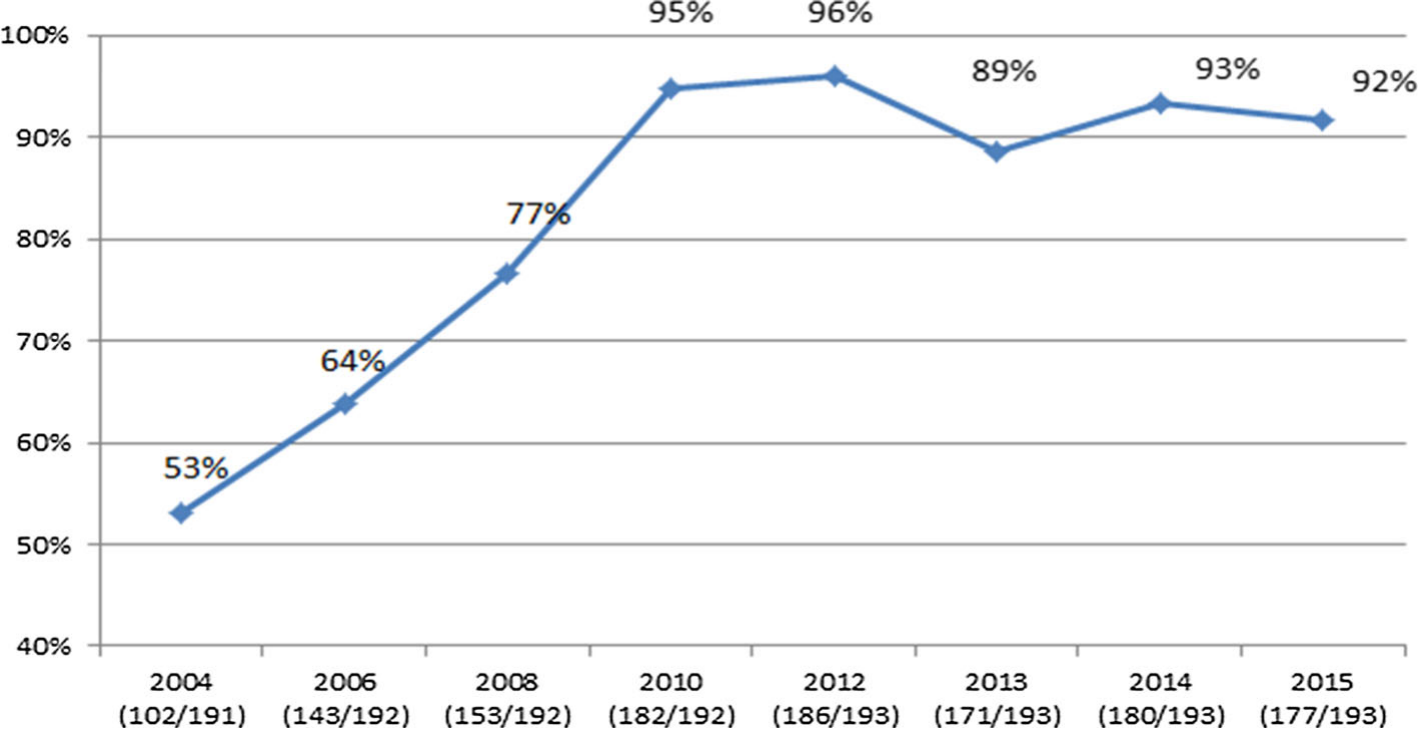
Global AIDS Reporting rates, 2004–2015. In the x-axis labels, for each reporting year, the number of countries/total number of United Nations member states is given in parenthesis

The reporting rate increased from 53% (52 Member States) in the 2004 round to a maximum of 96% (186 Member States) in the 2012 round and 92% (180 Member States) in the 2015 round (Fig. 2).

**Fig. 3:**
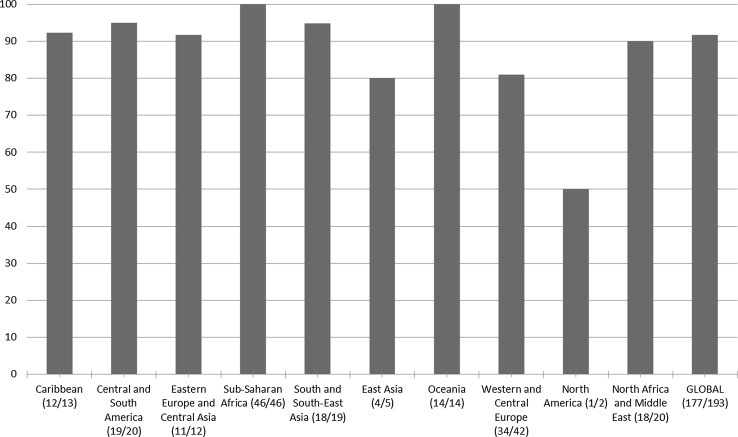
Proportion of countries that have participated in the 2015 Global AIDS Response Progress Reporting, by region

The reporting rates vary between different regions, with consistently very high rates in Sub-Saharan Africa, and lower reporting rates from Western Europe and North America (Fig. 3).

## Indicator Performance

**Fig. 4:**
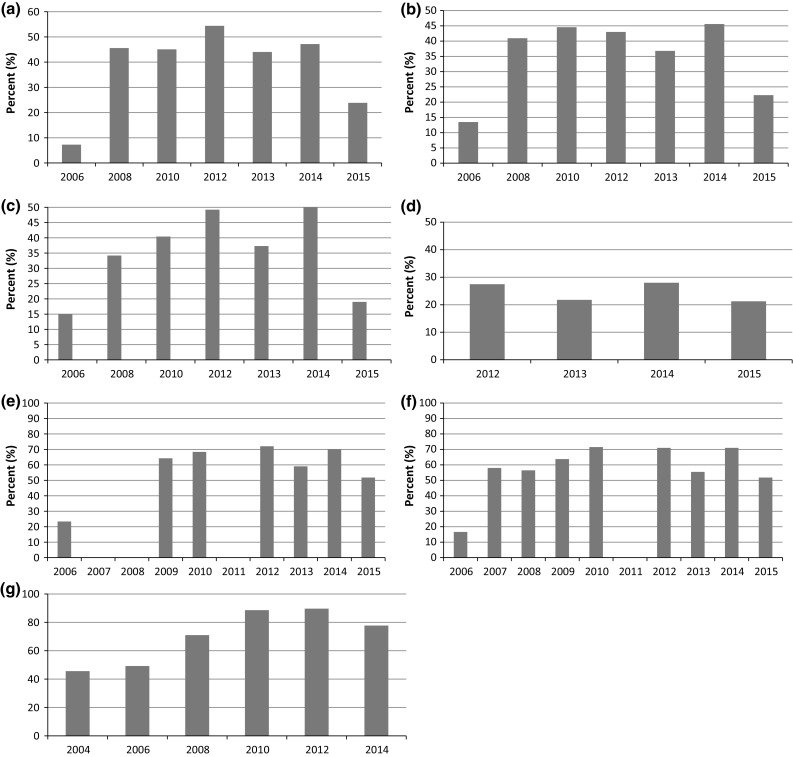
Reporting rates for key indicators in the Global AIDS Reporting 2006–2015 reporting rounds: **a** knowledge about HIV prevention (GARPR indicator 1.1), **b** sex workers: condom use (GARPR indicator 1.8), **c** men who have sex with men: condom use (GARPR indicator 1.12), **d** people who inject drugs: prevention programmes (GARPR indicator 2.1), **e** prevention of mother to child transmission (GARPR indicator 3.1), **f** antiretroviral therapy (GARPR indicator 4.1), and **g** the National Commitments and Policy Instrument (NCPI), Global reporting was every 2 years until the 2012 reporting round, except for the NCPI which has continued to be reported biennially, 2006–2015

The percent of countries reporting on the indicators that address knowledge about HIV prevention (GARPR indicator 1.1), sex workers: condom use (GARPR indicator 1.8), men who have sex with men: condom use (GARPR indicator 1.12), people who inject drugs: prevention programmes (GARPR indicator 2.1), prevention of mother to child transmission (GARPR indicator 3.1), antiretroviral therapy (GARPR indicator 4.1), and the National Commitments and Policy Instrument (NCPI) are presented in Fig. 4a–g.

Reporting rates increased rapidly 2006–2008 for indicators based on general population survey data (1.1) and key population survey data (1.8 and 1.12), most probably reflecting the roll-out of many general population surveys [such as Demographic and Health Survey (DHS)] and key population surveys [such as Integrated HIV Bio-Behavioral Surveillance (IBBS)]. For the indicator on condom use among men who have sex with men increasing reporting rates were seen until 2012, reaching close to 50%.

Indicator 2.1 measuring prevention programmes for people who inject drugs was revised from being based on selected survey data to a programmatic indicator in the 2012 reporting round, (as shown in Online Appendix for a full table of all UNGASS and GARPR indicators over time), therefore only reporting rates from 2012 onwards are available. This change was a result of the broader review of the UNGASS reporting system that occurred in 2010. The rationale behind the change was that programme data when reliable and informative showed a better picture of coverage and that it was easier to follow over time than survey based indicators.

Generally the programmatic indicators prevention of mother to child transmission (3.1) and antiretroviral therapy coverage (4.1) have had higher response rates than the survey based indicators, reaching maximum reporting rates of 70%.

In the 2015 reporting round, the language in the online reporting tool, used for countries to report the data on all indicators, was modified to indicate whether new data was available for each indicator, with a note to not enter indicator data that had been reported in previous years. Before the 2015 reporting round, countries were only asked whether data was available for the indicator, and previously reported data could be repeatedly reported (e.g. survey based data that are not collected every year). The decrease seen in the 2015 reporting round can most probably be explained by this. This change was introduced to reduce the burden for countries and to avoid repeated entry of the same data, which was leading to inappropriate interpretations in earlier years.

The NCPI response rates increased from 46 to 90% in 2012, with a slight decrease in 2014, more details about the development of the NCPI over time can be found in Torres et al. [18].

## Discussion

The Global AIDS reporting system has substantially improved over time and has provided key trend data on responses to the AIDS epidemic at global, regional and country level, serving as the global accountability mechanism and reference data for the global AIDS response.

Using data from the Global AIDS reporting system, it was recently reported that the MDG 6 on AIDS, “to halt and start to reverse the AIDS epidemic” has been achieved and that the goal of having 15 million people living with HIV on ART before the end of 2015 was reached nine months in advance of the target date [19]. This signals effective service delivery, monitoring mechanisms and accountability on the AIDS epidemic. This hopefully contributed to the high donor confidence, and highly successful Global Fund replenishment in 2016 which enables continued global efforts for 2017–2019.

In addition to the key characteristics of the Global AIDS Reporting system that have already been presented, multiple lessons have been learned as the global AIDS monitoring system evolved and improved. These lessons have provided insights as to specific factors that have contributed to building the Global AIDS Reporting System into a robust and sustainable strategic information system.

### A Standards-Based and Responsive Strategic Information System

The indicators in the indicator set have clearly been linked to targets contained in the political declarations, trying to keep the indicators to a limited set, but at the same time covering the larger spectrum of the AIDS response. The MERG, including key actors in the AIDS response, has served as the reference group whose role was to assure that the system has been standards based and responsive to the changing epidemic and response. User-friendly reporting guidance updated for each round, technical workshops, field adviser support, and financial support when needed have been other crucial components in building the system.

### Country Ownership and Broad Stakeholder Engagement (Including Civil Society)

The locus of control of the system has been at the country level, having national authorities leading the reporting process. The “three ones” highlighting the importance of having ONE M&E system as one of the three ones, the other two “ONEs” being One agreed HIV/AIDS Action Framework and One National AIDS Coordinating Authority was very important to get all partners to agree to harmonize indicators and work together with national authorities [20]. Further, the role of civil society has been instrumental in many countries, fulfilling both formal and informal monitoring functions [15].

### Commitment to Continuous Improvement at Global, Regional and Country Levels

As described, the Global AIDS Reporting System is built on a strong national ownership of reporting and is based on a broad consultative process across both government sectors and civil society. The development of the reporting system has required a combination of high-level political commitment and extensive technical collaboration between various elements of the UN system, national governments, bilateral and multilateral development agencies and civil society.

The continuous work to improve the system and change it when scientific or programmatic breakthroughs have led to new areas of work has also been important to keep the system up to date and relevant.

### Commitment to Transparency and Data Sharing

As shown above in text box 1 the data collected at global level has been shared as broadly as possible in reports and online, and through inclusion in other organisations’ databases. The over 35,000 annual individual visits to AIDSInfo illustrate broad need to access HIV data. The largest funding agents of the global AIDS response, the Global Fund and PEPFAR (Presidents Emergency Plan for AIDS Relief), use the country fact sheets and AIDSinfo data as part of their reference materials for grant applications, analysis, reports and data validation, comparing with their own programme data sets. A recently conducted survey on AIDSinfo (n = 347) showed that respondents use its data for the purposes of studies, research, HIV programming, advocacy, general knowledge and media reporting. The majority of UNAIDS publications reference the HIV related data collected through Global AIDS Reporting System. While sub-national level data is becoming increasingly available, UNAIDS has taken on the responsibility to also publish those data, which make data more meaningful for intervention design and HIV programming.

### A Focus on Usefulness at Country Level and on Sustainability

Both nationally and internationally, the Global AIDS Reporting has been viewed as more than just a reporting exercise to the UN General Assembly. The ultimate goal of the Global AIDS Reporting System has always been for national governments and their civil society partners to establish accountability mechanisms and to strategically use the data to inform their National Strategic Plans and guide more effective and sustainable responses to the HIV epidemic. The process has therefore emphasized country ownership of data and the onus of data collection, cleaning, validation, and aggregation rests with each reporting country.

The Global AIDS reporting process has important spin-offs in that it has catalyzed the development of national monitoring systems in many countries [21] and has greatly increased country level capacity for monitoring of the HIV epidemics and the response [22].

The indicators on AIDS spending and the policy environment have also been shown to be important components, which are missing in many other international reporting systems. The AIDS Spending indicator has made it possible to track how funds are spent at the national level and where the funds originate to help national decision-makers monitor whether funding allocations are in line with the specific country needs and help donors determine the return on their investment. The policy environment is monitored through the National Commitments and Policy Instrument (NCPI), the most comprehensive standardized global questionnaire available to assess the national policy, strategy, legal and programme implementation environment for the HIV response [18].

Furthermore, Global AIDS Reporting has been described as a non-binding legal instrument, which demonstrates that the use of a non-binding instrument can be remarkably effective in galvanizing increasingly deep commitments, action, reporting compliance and ultimately accountability for results [23].

## Challenges/Opportunities

The AIDS epidemic and the response are changing rapidly and the monitoring system must evolve with the changing context and environment.

The work to end the AIDS epidemic is primarily led at country level and the global monitoring and harmonization processes can exist only if they support country leadership and action. Therefore, a global monitoring system must facilitate national efforts, and avoid adding too much of a burden on national systems. High quality global data is essential for global accountability and tracking of the epidemic. Balancing between globally significant indicators, and those that focus on national programmatic accountability can be a trade-off, and it is important that there be an ongoing dialogue to balance the needs of global and national interests. Further, some indicators might be of different relevance in e.g. low-income countries and high-income countries, adding to the complexity of trying to maintain one global, yet nationally relevant monitoring framework.

The launch of the MDGs and the UNGASS reporting system encouraged partners at global, regional and national level to build M&E systems and improve them over time. The last decade has seen increased commitments and spending on HIV M&E, as well as improved M&E capacity [22]. However, while these systems will soon celebrate 15 years, can this interest and commitment be maintained? Not only do monitoring systems need routine maintenance, but also continued inspiration, enthusiasm and a sense of purpose for its key stakeholders. These systems must continue to prove their value to key users, and the audiences they serve.

Some areas have proven to be more challenging than others to monitor, e.g. hard to reach populations, such as sex workers, men who have sex with men and people who inject drugs [24].

Many countries are improving both the collection and the use of data at the subnational level to better understand the epidemic and the response. Such data will help all stakeholders to better understand the geographic distribution of HIV epidemics and the responses at community level [25]. For a few indicators, the Global AIDS reporting system [13] has made provision for sub-national level data, which was submitted for the first time in 2015. This shift is in-line with the United Nations Secretary-General’s Independent Expert Advisory Group on a Data Revolution for Sustainable Development (IEAG) and its views on data revolution. UNAIDS vision on future global AIDS data is aiming to produce data with greater frequency, detail to location, and relevance to programmes which will be key elements of measurement towards ending the AIDS epidemic by 2030. In addition, as the world becomes more focused on the cost-effectiveness and efficiencies of programmes, new indicators will be needed to monitor these dimensions.

## Conclusion

The Global AIDS reporting system will be critical in supporting the post-2015 monitoring of the AIDS response through the Sustainable Development Goals in view of ending the AIDS epidemic as a public health threat by 2030. Many of the challenges, obstacles and biases that arose during the evolution of the monitoring systems for the AIDS response were not unique to the AIDS epidemic. However, it was among the first monitoring frameworks in health to showcase how political, social, financial, behavioral, and service delivery indicators can complement each other. While governments often may resist external pressures for implementing such mechanisms, the Global AIDS Response Progress Reporting has demonstrated that the information that is produced can remain of high quality, even with decreasing external support, once it has proven its added value in accountability. Such mechanisms may arise in new areas of health delivery, driven by current crises, (such as Ebola, Zika and others), and as the global community moves into new areas of health services, including Universal Health Coverage, Non Communicable Diseases, antibiotic resistance, etc. AIDS progress reporting can help in understanding the need for integration between different monitoring systems, data sources, and the ongoing dialogue that must be generated between different sector experts.

## Electronic supplementary material

Below is the link to the electronic supplementary material.

Appendix (UNGASS and GARPR Indicators Summary table) (XLSX 21 kb)
